# Syndecan-4 functionalization of tissue regeneration scaffolds improves interaction with endothelial progenitor cells

**DOI:** 10.1093/rb/rbab070

**Published:** 2021-11-29

**Authors:** Harleigh Warner, Yidi Wu, William D Wagner

**Affiliations:** 1 Department of Plastic and Reconstructive Surgery, Wake Forest University School of Medicine, Medical Center Blvd., Winston-Salem, North Carolina 27157, USA; 2 Department of Biomedical Engineering, Wake Forest University School of Biomedical Engineering and Sciences, Medical Center Blvd., Winston-Salem, North Carolina 27157, USA

**Keywords:** regenerative scaffolds, syndecan-4, stromal derived factor-1α, endothelial progenitor cells, cardiovascular scaffolds

## Abstract

Key to most implanted cell free scaffolds for tissue regeneration is the ability to sequester and retain undifferentiated mesenchymal stem cells at the repair site. In this report, syndecan-4, a heparan sulfate containing proteoglycan, was investigated as a unique molecule for use in scaffold functionalization. An electrospun hybrid scaffold comprised of poly (glycerol) sebacate (PGS), silk fibroin and type I collagen (PFC) was used as a model scaffold to develop a procedure and test the hypothesis that functionalization would result in increased scaffold binding of endothelial progenitor cells (EPCs). For these studies both Syndecan-4 and stromal derived factor-1α (SDF-1α) were used in functionalization PFC. Syndecan-4 functionalized PFC bound 4.8 fold more SDF-1α compared to nonfunctionalized PFC. Binding was specific as determined by heparin displacement studies. After culture for 7 days, significantly, more EPCs were detected on PFC scaffolds having both syndecan-4 and SDF-1α compared to scaffolds of PFC with only syndecan-4, or PFC adsorbed with SDF-1α, or PFC alone. Taken together, this study demonstrates that EPCs can be bound to and significantly expanded on PFC material through syndecan-4 mediated growth factor binding. Syndecan-4 with a multiplicity of binding sites has the potential to functionalize and expand stem cells on a variety of scaffold materials for use in tissue regeneration.

## Introduction

Numerous bioengineered scaffolds intended for use in tissue regeneration lack cells but incorporate secondary modifications designed to attract stem cells post implantation. While this is important in all types of scaffolds, it is highly significant for cardiovascular scaffolds where rapid endothelization provides a nonthrombogenic surface.

In this study, the use of a heparan sulfate containing proteoglycan, syndecan-4, was investigated as a possible binding site for SDF-1α in order to attract CD34+ endothelial progenitor cells (EPCs) to an endovascular scaffold termed, PFC [1]. Syndecans are single pass transmembrane proteoglycans consisting of a core protein with pendant heparan sulfate glycosaminoglycan side chains [2]. As an intact transmembrane protein, syndecan-4 is involved in signaling pathways including cellular proliferation, migration, mechanotransduction, and endocytosis [3]. The extracellular domain of syndecan-4 includes pendant heparan sulfate chains, which have been shown to facilitate the binding of SDF-1α to the CXCR4 receptor [4]. Unlike heparin, syndecan-4 is a normal component of endothelial and smooth muscle cell surfaces and has a larger and more complex structure with heparan sulfates existing in a spatially extended and stable conformation. This proteoglycan is therefore an ideal choice for engineered scaffold functionalization.

PFC is an electrospun composite of poly (glycerol-sebacate) (PGS), silk fibroin, and type I collagen. The viscoelastic material is mechanically robust, has minimal degradation *in vitro*, supports endothelial monolayer formation and has low thrombogenic potential [1]. Thus, PFC is an ideal scaffold to use as a model biomaterial for secondary modification with syndecan-4.

Stromal derived factor-1α (SDF-1α)/CXCL12 is a cytokine that has been shown to localize EPCs to areas of ischemia through the CXCR4 signaling pathway [5]. Currently used methods to bind SDF-1α to vascular grafts include functionalization with heparin followed by incubating the grafts in SDF-1α [6–8]; coating the grafts with fibronectin and then incubating the grafts in SDF-1α [9, 10]; and coating grafts in a heparin coacervate doped with SDF-1α [11]. While these procedures have shown promise in binding SDF-1α, non-covalent coatings are typically non-resilient to vascular flow. In this study, an improved protocol using a heparan sulfate proteoglycan (syndecan-4) was designed and tested in order to investigate the ability to bind SDF-1α and interact with EPCs.

**Figure 1.**
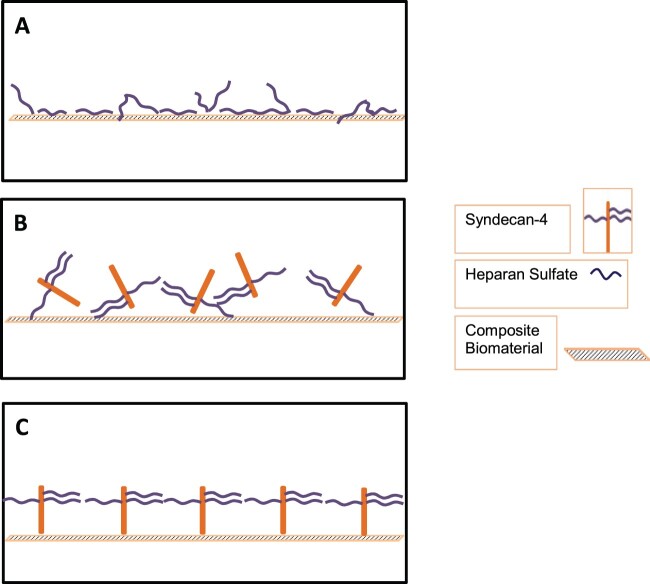
Schematic illustrating binding of syndecan-4 to PFC. (**A**) One-step NHS/EDC conjugation with carboxylic acid containing heparan sulfate chains and an amine containing biomaterial. (**B**) One-step NHS/EDC conjugation with carboxylic acid containing heparan sulfate chain on syndecan-4 and an amine containing biomaterial. (**C**) Two-step NHS/EDC conjugation with amine containing core protein of syndecan-4 and a carboxylic acid containing biomaterial

A common method of functionalizing heparin and heparan sulfate or oligosaccharides to biomaterials involves functionalizing the carboxylic acid group, and conjugation to an amine group of the biomaterial [6, 12, 13]. This one-step addition scheme of adding an amine containing biomaterial to a solution of MES buffer with NHS/EDC and heparin/heparan sulfate may result in nonspecific covalent linkages (Fig. 1A). Linkages may occur between the amine groups present on the biomaterial with the carboxylic acid groups on heparan sulfate, linkages between carboxylic acid groups present on the biomaterial with the amine groups on the protein chain, as well as linkages within and between heparan sulfate chains. This nonspecific binding may block the binding sites on heparan sulfate chains of syndecan-4 (Fig. 1B). In this study, to prevent this occurrence, a two-step procedure was used where PFC was exposed first to NHS/EDC in order to bind to the carboxylic acid groups on the biomaterial. After rinsing the material, adding syndecan-4 permitted only the amine groups present on the biomaterial to covalently link with the EDC thus preventing any binding within the heparan sulfate chains. This maintains heparan sulfate in a pendant state and available to bind growth factors (Fig. 1C). In the present study, syndecan-4 was used to functionalilze PFC to immobilize SDF-1α and successfully resulted in attachment of EPCs.

## Materials and methods

### Materials

Silk fibroin protein was extracted from raw silk (Haian Silk Company, Nantong, China) using an aqueous solvent processing method according to published protocols [14]. PGS prepolymer was synthesized from glycerol (Fisher Scientific) and sebacic acid (Sigma-Aldrich) following published methods [15]. Type I collagen from calfskin was purchased commercially (Elastin Products Company, Inc.). Recombinant human syndecan-4 and human CXCL12/SDF-1α were purchased from R&D Systems.

### Electrospinning of PFC fibers

Electrospun solutions were prepared using type I collagen, silk fibroin, and PGS at a mass ratio of 4.5:4.5:1 and were dissolved in 1, 1, 1, 3, 3, 3-hexafluoro-2-propanol (HFIP) (Sigma-Aldrich) at a 10% w/v ratio. The solution was loaded into a 5 ml syringe affixed to infusion pump (Baxter, AS50) and ejected from an 18-gauge blunt tip needle at a rate of 2 ml/hr. A voltage of 25 kV was applied at a distance of 18 cm between the syringe tip and the collector plate. A total of 3.5 ml of solution resulted in scaffolds having a thickness of 1 mm. The electrospun scaffold was removed from collector plate and heated at 120°C for 48 hours to heat polymerize the PGS component. The material was then exposed to 1.5% glutaraldehyde vapor (Sigma-Aldrich) for 24 hours and subsequently washed in 0.02 M glycine solution for 1 hour to block unreacted aldehydes.

### Covalent linkage of syndecan-4 to PFC

PFC scaffolds were cut to fit to 96 well plates and either adsorbed or covalently linked with syndecan-4. For adsorption, material was incubated with 0.8 µg syndecan-4/cm^2^ PFC in PBS for 2 hours at 37°C with agitation. For covalently binding, a two-step NHS/EDC conjugation was used [16]. PFC was incubated in 2-morpholinoethane sulfonic acid (MES) (Sigma-Aldrich) buffer (0.5 M NaCl, 0.1 M MES, pH 6) for 30 minutes at room temperature. The material was then incubated in MES buffer with 30 mmol 1-ethyl-3-(3-dimethylaminopropyl) carbodiimide (EDC) (Thermo Scientific) and 6 mmol N-hydroxysuccinimide (NHS) (Sigma Aldrich) for 30 minutes at room temperature followed by rinsing with MES buffer to activate the carboxyl groups present on the PFC. The material was then incubated in 0.8 µg syndecan-4/cm^2^ PFC in PBS for 2 hours at 37°C with agitation. After this reaction, PFC was washed with 0.1 M Na_2_HPO_4_ and PBS (3 times each) to deactivate unreacted EDC. PFC with covalently bound syndecan-4 is referred to as PFC_SYN_ throughout the manuscript.

### Detection of bound syndecan-4

PFC and PFC_SYN_ (*n* = 4) were added to wells of a 96 well plate, rinsed with PBS, and blocked with 0.1% casein (Sigma Aldrich) in PBST (PBS + 0.05% Tween-20) overnight at 4°C. Samples were then incubated with primary antibody mouse IgG anti-syndecan-4 (Santa Cruz) (1:1000) diluted in 0.1% casein in PBST for 2 hours at room temperature. Samples then were rinsed with PBS with 0.05% Tween-20 and incubated with HRP—anti mouse IgG secondary antibody (Abcam) (1:10 000) diluted in 0.1% casein in PBST. HRP substrate 3,3′,5,5′-tetramethylbenzidine (TMB) (Thermo Scientific) was added for 30 minutes and the reaction was quenched with 2 M sulfuric acid (Sigma-Aldrich). PFC and PFC_SYN_ materials were removed from wells and the absorbance of the remaining solution was read at 450 nm using a plate reader.

### Saturation kinetics of syndecan-4 binding to PFC

To determine the saturation level of syndecan-4, increasing amounts of syndecan-4 were incubated with PFC during functionalization. The amount of syndecan-4 was determined by ELISA as described previously. Saturation kinetics was determined by plotting ELISA data as a Langmuir adsorption isotherm. Assumptions were that (1) syndecan-4 had a high affinity to the PFC (2) the PFC surface had a specific number of sites where the solute molecules could be adsorbed (3) the adsorption involved only one monolayer of syndecan-4 on the PFC.

### Morphology of electrospun fibers

A scanning electron microscope (JEOL) was used to determine fiber structure of PFC, PFC_SYN_, and PFC after exposure to NHS/EDC. Fibers were attached to sample mounts using carbon tape and sputter coated with gold for image analysis. All micrographs were acquired under the same magnification, working distance, and electron beam density.

### SDF-1α + syndecan-4 ELISA

PFC_SYN_ was fabricated with 0.8 µg syndecan-4/cm^2^ as described previously. After PFC and PFC_SYN_ (*n* = 3) was rinsed in Na_2_HPO_4_ and PBS, it was added to a PBS solution containing 0.8 µg SDF-1α/cm^2^ PFC. PFC and PFC_SYN_ were incubated with SDF-1α for 2 hours at 37°C with agitation followed by rinsing 3 times with PBS. To determine saturation kinetics of SDF-1α binding to PFC_SYN_, material was incubated with increasing concentrations of SDF-1α. Amounts of SDF-1α were determined by ELISA as described previously, using mouse IgG anti-SDF-1α (1:100) (R&D Systems) as the primary antibody and HRP anti- mouse IgG (Abcam) (1:10 000) as the secondary antibody.

### Heparin displacement study

This experiment was done to demonstrate that SDF-1α binding was selective for syndecan-4. For these studies, the ionic interaction was displaced with increasing concentrations of highly sulfated heparin. In this experiment, PFC_SYN_ was fabricated with 0.8 µg syndecan-4/cm^2^ PFC as described previously, rinsed with Na_2_HPO_4_ and PBS, and then incubated with 0.8 µg SDF-1α/cm^2^ PFC_SYN_ for 2 hours at 37°C with agitation. After washing with PBS for 3 times, the scaffolds were incubated with increasing amounts of porcine intestinal mucosa heparin (Fresenius Kabi) at 0, 1, 10, 50, 75, 100, or 200 µg heparin/cm^2^ PFC_SYN_ in PBS at room temperature with agitation for 3 hours. The scaffolds were washed 3 times with PBS after incubation and the remaining amount of SDF-1α on the materials was quantified by ELISA as described previously. Mouse IgG anti-SDF-1 (R&D Systems) (1:100 dilution) was used as the primary antibody and HRP anti-mouse IgG (Abcam) (1:10 000 dilution) was used as the secondary antibody. The amount of SDF-1α on PFC_SYN_ quantified with ELISA was normalized by the absorbance of control group (no heparin added) and plotted against the heparin concentration.

### Cell culture

Human Bone Marrow Derived cells (PCS-800-012) were purchased from ATCC. Cells were characterized by flow cytometry to be positive for CD34 and CD45. Cells were cultured on fibronectin (Sigma-Aldrich) coated (1 µg/cm^2^) flasks using complete Endothelial Cell Basal Media (ATCC). The media was supplemented with Endothelial Cell Growth Kit VEGF (ATCC) (without hydrocortisone), supplemented with 10% FBS and 1% penicillin-streptomycin. After 4 days, non-adherent cells were removed. At 7 days, cell colonies appeared. Cells were trypsinized and replated to distribute colonies. Cells were subcultured at 80% confluence.

### Cell characterization

The CXCR4 receptor was identified on cells by immunofluorescence staining. Cells were plated in a fibronectin coated 24 well plate at a density of 5000 cells/cm^2^ and incubated in 500 μl culture medium for 24 hours at 37°C. Cells were fixed in 4% paraformaldehyde for 40 minutes, blocked with 1% BSA overnight at 4°C, incubated with rabbit anti-CXCR4 (Thermo Scientific) (1:200) primary antibody for 45 minutes at 37°C, and incubated with AlexaFluor 488 goat anti-rabbit IgG (Abcam) (1:400) secondary antibody for 45 minutes at 37°C. Nuclei were counterstained with DAPI.

### Cell attachment assay

CD34+ cells were suspended in basal media + 1% penicillin-streptomycin and 10 000 cells were seeded in low attachment 24 well plates. Scaffolds (PFC, PFC_SYN_, PFC/SDF-1α, and PFC_SYN_/SDF-1α) were added to wells (*n* = 4). Syndecan-4 was added to material at 0.8 µg syndecan-4/cm^2^ PFC. SDF-1α was added to material at 250 ng/cm^2^ PFC. All well plates were places on an orbital shaker in a 37°C incubator and rotated at 4 rpm to avoid cell aggregation. On days 4 and 7 material was removed from culture weighed and analyzed for DNA content. For these studies, cells were cultured in serum free media to minimize any effects of cytokines.

### Fibroblast attachment and growth

Selectivity for cells having receptors for SDF-1α was evaluated by comparing EPC and fibroblast interaction with scaffolds. PFC and PFC_SYN_/SDF-1α materials were added to ultra-low attachment plates (*n* = 3), PFC_SYN_/SDF-1α was fabricated as described previously. EPCs or fibroblasts (no CXCR4 receptors) were added to the materials at 10 000 cells per well, on day 4 PFC and PFC_SYN_/SDF-1α materials were removed from culture and the number of EPCs or fibroblasts were quantified by DNA measurement.

### DNA analysis

Scaffolds (PFC, PFC_SYN_, PFC/SDF-1α, and PFC_SYN_/SDF-1α) were placed in 250 µL cell lysis solution (0.2% v/v Triton X-100, 10 mM Tris pH 7.0, and 1 mM EDTA) for 30 minutes on ice and vortexed 3 times for 10 seconds each. Cell lysis solution was placed in 1.5 ml tubes and double stranded DNA content of lysates was quantified with Quant-iT™ PicoGreen^®^ dsDNA kit (Invitrogen) according to manufacturer’s instructions. Briefly, cell lysate was diluted 10x in TE buffer. DNA standards were diluted in 9:1 (TE Buffer: cell lysis solution). Samples and standards (100 µl) were added to a black bottom 96 well plate. PicoGreen^®^ reagent (100 µl) was added to each of the wells. The plate was covered with foil and mixed on a shaker plate for 5 minutes. The plate was read at excitation: 460 nm, emission: 540 nm. The amount of DNA was calculated from the standard curve and normalized to wet weight of material.

### Statistical analysis

All quantitative data were presented as mean ± standard error of the mean (SEM). GraphPad Prism software (Version 9.2.0) was used for statistical analysis. Statistical comparisons were determined by Student’s *t*-test for studies with two comparisons or analysis of variance for studies with multiple comparisons. If significant effects were determined, a Tukey’s *post hoc* test was utilized to identify significant differences between individual means. Results of *P* < 0.05 were considered significant.

## Results

### Functionalization of PFC with syndecan-4

**Figure 2.**
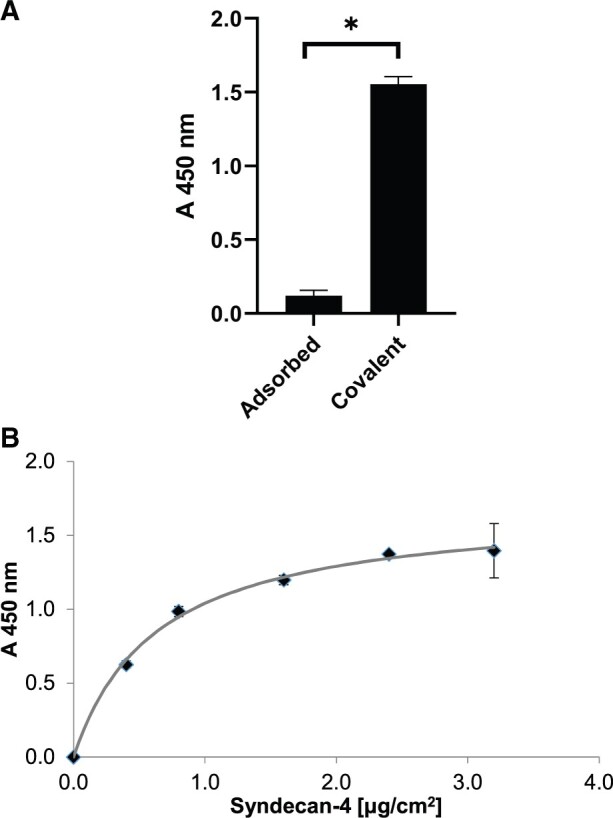
(**A**) PFC with adsorbed or covalently linked syndecan-4. Bars represent means and lines represent SEM. (**B**) Saturation curve for covalently bound syndecan-4 on PFC (*N* = 3 observations for syndecan-4 concentrations). SEM ranged from 0.02–0.03 for syndecan-4 levels from 0.40 to 2.40 µg/cm^2^ (*P < 0.001)

In an initial experiment, PFC was functionalized with syndecan-4 via passive adsorption or by covalent binding by using carbodiimide crosslinking chemistry. Twelve fold more syndecan-4 was detected by ELISA on PFC after covalent linkage compared to PFC with adsorbed syndecan-4 (*P* < 0.001) (Fig. 2A). In a further experiment, the saturation of covalently bound syndecan-4 to PFC was determined using increasing concentrations of syndecan-4. Saturation was calculated to be 0.8 µg syndecan-4/cm^2^ PFC (Fig. 2B). This concentration of syndecan-4 was used for further experiments involving SDF-1α binding.

**Figure 3.**
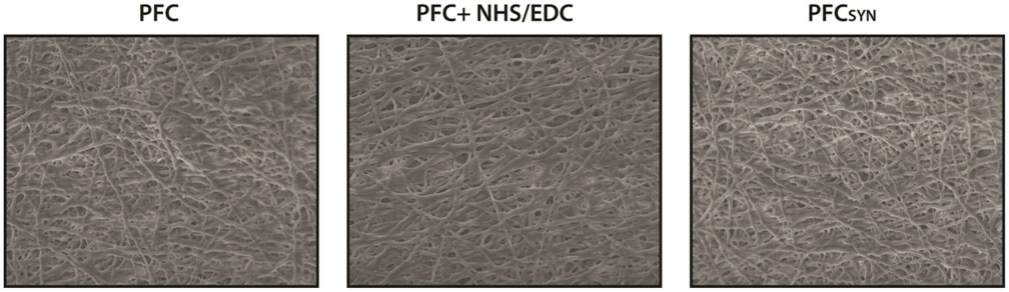
Scanning electron micrographs of PFC scaffolds crosslinked with 1.5% glutaraldehyde and subsequently treated with NHS/EDC or functionalized with syndecan-4 (PFC_SYN_)

PFC was imaged using scanning electron microscopy at several magnifications to determine if covalently bound syndecan-4 modified fiber structure. Covalently adding syndecan-4 or exposing material to NHS/EDC did not affect fiber morphology (Fig. 3). At high magnification, no fiber surface modifications such as erosive pitting, blistering or blebbing were observed between treatments.

### Binding of SDF-1α to PFC_SYN_

**Figure 4.**
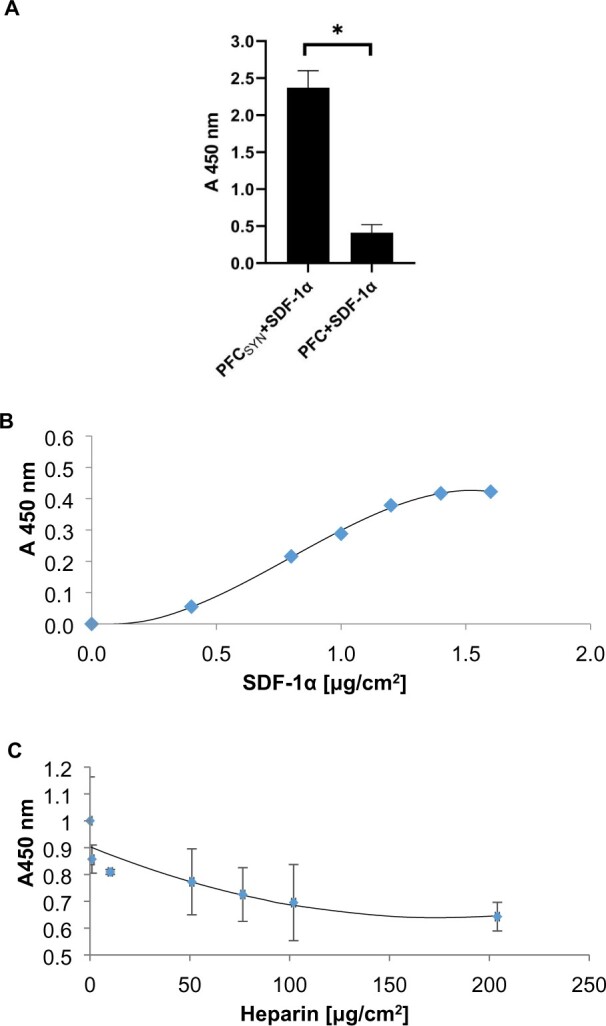
(**A**) SDF-1α bound to PFC with and without syndecan-4. Bars represent means and lines represent SEM. (**B**) Saturation kinetics of SDF-1α on PFC_SYN_. (**C**) Displacement of SDF-1α from PFC_SYN_ by heparin (*P < 0.001)

In an initial experiment, SDF-1α binding to PFC_SYN_ was evaluated and compared to non-functionalized PFC. A 4.7 fold increase in SDF-1α was detected in PFC conjugated with syndecan-4 compared to PFC without syndecan-4 (*P* < 0.001) (Fig. 4A). Next, the saturation of SDF-1α on PFC_SYN_ was determined. For this experiment, PFC was conjugated with 0.8 µg syndecan-4/cm^2^ prior to binding with increasing concentrations of SDF-1α. Saturation of SDF-1α on PFC_SYN_ occurred at 1.2 µg SDF-1α/cm^2^ (Fig. 4B).

### *Specificity of SDF-1*α *binding to PFC_SYN_*

The specific binding of SDF-1α to syndecan-4 was determined by using heparin to displace SDF-1α. The results demonstrated that the amount of SDF-1α remaining on the material decreased as the heparin concentration increased. Displacement plateaued around 150 μg heparin/cm^2^ PFC_SYN_ (Fig. 4C).

### Interaction of EPC with PFC functionalized with syndecan-4 and SDF-1α

Since the receptor for SDF-1α is CXCR4, EPCs were evaluated first for the presence of the receptor. Fluorescent images demonstrated that cells throughout the culture were positive for the CXCR4 receptor (see Supplementary Fig. 1).

**Figure 5.**
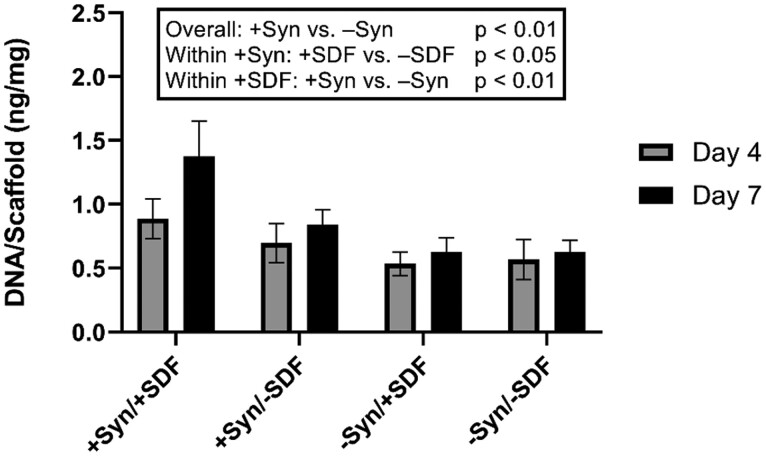
Quantification of EPCs on PFC scaffolds with syndecan-4 (Syn) and SDF-1α (SDF). Bars represent means and lines represent SEM

These cells were used to determine the interaction with functionalized PFC using an *in vitro* adhesion assay. Published work using grafts constructed of PGS demonstrated that bioactivity and EPC homing occurred at concentrations of 100 ng SDF-1α/µL [11]; therefore, 250 ng SDF-1α/cm^2^ PFC was used to treat the material. There was a significant increase EPCs on PFC_SYN_ as compared to PFC without syndecan-4 (*P* < 0.01). Within the sub-group of PFC functionalized with syndecan-4, there was a significant increase in cells on PFC_SYN_ having SDF-1α as compared to PFC_SYN_ without SDF-1α (*P* < 0.05). In the subgroup of PFC having SDF-1α, a significant increase in cells was observed for PFC functionalized with syndecan-4 as compared to PFC without syndecan-4 (*P* < 0.01) (Fig. 5).

**Figure 6.**
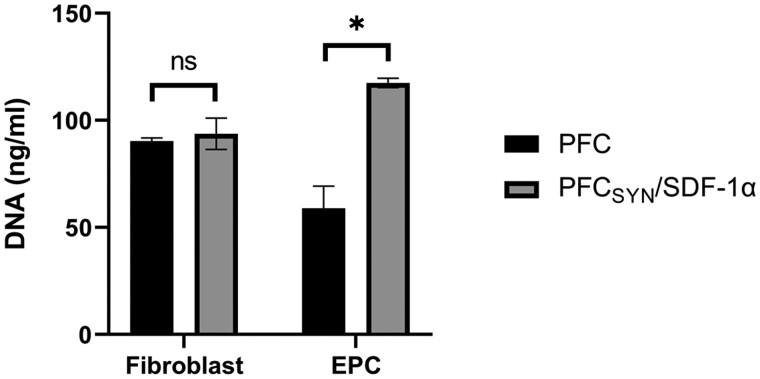
Quantification of fibroblasts and EPCs on PFC with syndecan-4 (SYN) and SDF-1α. Bars represent means and lines represent SEM (*P < 0.01)

In order to demonstrate PFC_SYN_/SDF-1α specifically bound EPCs with SDF-1α and not cells without receptors, fibroblasts were examined in an additional study. Compared to fibroblasts, the numbers of EPCs were significantly (*P* < 0.01) increased by 100% on PFC functionalized with syndecan-4 and SDF-1α whereas no increases were seen for fibroblasts (Fig. 6).

## Discussion

The present study was designed to demonstrate that syndecan-4, a heparan sulfate proteoglycan, could be used to attract EPCs to scaffolds used for tissue regeneration. Through a series of experiments, PFC, a hybrid elastomeric material, was functionalized covalently with syndecan-4, and ionically with SDF-1α. The covalent addition of syndecan-4 to PFC did not modify the morphology of the PFC scaffold. Addition of syndecan-4 demonstrated saturation kinetics and at maximum saturation, significant binding of SDF-1α was observed. This binding was associated with heparan sulfate on syndecan-4 as evidenced by displacement of heparin. Experiments using EPCs demonstrated that the presence of syndecan-4 and SDF-1α on PFC resulted in significant increased binding of cells. This binding was attributed to the presence of CXCR4 receptors on EPCs, since no increased binding was observed using fibroblasts lacking CXCR4 receptors.

In this study, NHS/EDC chemistry was used to link the carboxylic acid groups present in collagen and fibroin of PFC to the amines present in the protein core of syndecan-4. In the protocol, NHS facilitates the addition of EDC to carboxylic acid groups. Upon exposure to an amine group, EDC links the carboxylic acid group to the amine group [16]. This method of conjugation may result in interchain linkage as well as the blocking of binding sites present on the heparin. The protein core of syndecan-4 contains significant amounts of primary amines whereas, heparan sulfate has minimal unsubstituted amine groups [17]. The two-step protocol was used and thus resulted in only the amine groups present on syndecan-4 core protein to covalently link with the EDC, thus was successful in preventing activation of carboxyl groups on heparan sulfate of syndecan-4.

Compared to the use of heparin, functionalization with syndecan-4 represents an improved method of immobilizing growth factors due to its ubiquitous presence and function in native tissue, molecular size, molecular conformation, and more varied oligosaccharide structure. A limitation of functionalizing biomaterials with heparin or only the heparan sulfate moiety of syndecan-4, is that the covalent linkage may impact the structure and therefore function of the carbohydrate [18, 19]. By using larger molecules such as intact syndecan-4 for functionalization, specific domains on the protein can be targeted for covalent linkage, while leaving the pendant heparan sulfate side chains unaffected and accessible for molecular interactions. Compared with non-covalent functionalization such as adsorption, the covalent linkage of syndecan-4 and PFC will provide a higher binding strength that is more resilient to vascular flow. The binding affinity of SDF-1α to heparan sulfate has been reported to be 30 nM, which indicates high binding strength to sydecan-4 [20].

Perlecan, another heparan sulfate containing proteoglycan also may be used in cardiovascular scaffold functionalization. Perlecan has been shown to support endothelial cell adhesion and growth while inhibiting platelets and smooth muscle cell interaction [21]. However, the size and molecular domain of perlecan is much greater than syndecan-4. Thus, functionalization with perlecan may be bulky and distanced from the surface of scaffold material.

This study is novel in that it utilizes syndecan-4 to immobilize SDF-1α. We have shown that it is possible to link covalently syndecan-4 to PFC, a composite elastomeric endovascular biomaterial. Additionally, we were able to immobilize SDF-1α to syndecan-4 on PFC and increase the binding and growth of EPCs on this scaffold. The multifunctionality residing in the variable and specific oligosaccharide sequence of heparan sulfate of syndecan-4 also may provide high affinity binding potential for sequestration of other types of growth factors in circulation or produced in a paracrine manner. Although only PFC was investigated in this study, a broader application includes the use of syndecan-4 to functionalize a variety of composite biomaterials.

## Conclusion

The goal of the study was to functionalize a composite biomaterial, PFC, in order to bind EPCs to a scaffold material. The results of the study demonstrated that syndecan-4 can be linked covalently to PFC while maintaining heparan sulfates available for subsequent molecular interactions. The functionalization provided a significant increase above non-functionalized PFC in the ability to sequester the growth factor, SDF-1α. The study provides a procedure whereby SDF-1α as well as other growth factors can be bound to a variety of scaffolds through use of syndecan-4. The findings of this study provide useful information for the rational design of protocols for harboring growth factors on various type of biomaterials with the eventual outcome to provide enhanced retention of undifferentiated progenitor cells necessary for tissue regeneration.

## Supplementary data

Supplementary data are available at *REGBIO* online.

## Supplementary Material

rbab070_Supplementary_Data

## References

[1] Wang R , Levi-Polyanchenko N, Morykwas M et al Novel nanofiber-based material for endovascular scaffolds. J Biomed Mater Res A 2015;103:1150–8. 25044469 10.1002/jbm.a.35267

[2] Bernfield M , Götte M, Park PW et al Functions of cell surface heparan sulfate proteoglycans. Annu Rev Biochem 1999;68:729–77. 10872465 10.1146/annurev.biochem.68.1.729

[3] Carey DJ. Syndecans: multifunctional cell-surface co-receptors. Biochem J 1997;327:1–16. 9355727 10.1042/bj3270001 PMC1218755

[4] Charnaux N , Brule S, Hamon M et al Syndecan-4 is a signaling molecule for stromal cell-derived factor-1 (SDF-1)/CXCL12. FEBS J 2005;272:1937–51. 15819887 10.1111/j.1742-4658.2005.04624.x

[5] De Falco E , Porcelli D, Torella AR et al SDF-1 involvement in endothelial phenotype and ischemia-induced recruitment of bone marrow progenitor cells. Blood 2004;104:3472–82. 15284120 10.1182/blood-2003-12-4423

[6] Yu J , Wang A, Tang Z et al The effect of stromal cell-derived factor-1α/heparin coating of biodegradable vascular grafts on the recruitment of both endothelial and smooth muscle progenitor cells for accelerated regeneration. Biomaterials 2012;33:8062–74. 22884813 10.1016/j.biomaterials.2012.07.042 PMC3488434

[7] Shafiq M , Kong D, Kim SH. SDF-1α peptide tethered polyester facilitates tissue repair by endogenous cell mobilization and recruitment. J Biomed Mater Res A 2017;105:2670–84. 28571106 10.1002/jbm.a.36130

[8] Zhou J , Ye X, Wang Z et al Development of decellularized aortic valvular conduit coated by heparin–SDF-1α multilayer. Ann Thorac Surg 2015;99:612–8. 25499473 10.1016/j.athoracsur.2014.09.001

[9] De Visscher G , Mesure L, Meuris B et al Improved endothelialization and reduced thrombosis by coating a synthetic vascular graft with fibronectin and stem cell homing factor SDF-1α. Acta Biomater 2012;8:1330–8. 21964214 10.1016/j.actbio.2011.09.016

[10] Flameng W , De Visscher G, Mesure L et al Coating with fibronectin and stromal cell–derived factor-1α of decellularized homografts used for right ventricular outflow tract reconstruction eliminates immune response–related degeneration. J Thorac Cardiovasc Surg 2014;147:1398–404. 23896322 10.1016/j.jtcvs.2013.06.022

[11] Lee K-W , Johnson NR, Gao J et al Human progenitor cell recruitment via SDF-1α coacervate-laden PGS vascular grafts. Biomaterials 2013;34:9877–85. 24060423 10.1016/j.biomaterials.2013.08.082 PMC3882008

[12] Kang I-K , Kwon OH, Lee YM et al Preparation and surface characterization of functional group-grafted and heparin-immobilized polyurethanes by plasma glow discharge. Biomaterials 1996;17:841–7. 8730969 10.1016/0142-9612(96)81422-0

[13] Lee J , Yoo J, Atala A et al Controlled heparin conjugation on electrospun poly (ε-caprolactone)/gelatin fibers for morphology-dependent protein delivery and enhanced cellular affinity. Acta Biomater 2012;8:2549–58. 22465575 10.1016/j.actbio.2012.03.030

[14] Rockwood DN , Preda RC, Yücel T et al Materials fabrication from Bombyx mori silk fibroin. Nat Protoc 2011;6:1612–31. 21959241 10.1038/nprot.2011.379 PMC3808976

[15] Wang Y , Ameer GA, Sheppard BJ et al A tough biodegradable elastomer. Nat Biotechnol 2002;20:602–6. 12042865 10.1038/nbt0602-602

[16] Nakajima N , Ikada Y. Mechanism of amide formation by carbodiimide for bioconjugation in aqueous media. Bioconjug Chem 1995;6:123–30. 7711098 10.1021/bc00031a015

[17] Toida T , Yoshida H, Toyoda H et al Structural differences and the presence of unsubstituted amino groups in heparan sulphates from different tissues and species. Biochem J 1997;322:499–06. 9065769 10.1042/bj3220499 PMC1218218

[18] Osmond RI , Kett WC, Skett SE et al Protein–heparin interactions measured by BIAcore 2000 are affected by the method of heparin immobilization. Anal Biochem 2002;310:199–207. 12423639 10.1016/s0003-2697(02)00396-2

[19] Powell AK , Yates EA, Fernig DG et al Interactions of heparin/heparan sulfate with proteins: appraisal of structural factors and experimental approaches. Glycobiology 2004;14:17R–30R. 10.1093/glycob/cwh051 14718374

[20] Xu D , Esko JD. Demystifying heparan sulfate–protein interactions. Annu Rev Biochem 2014;83:129–57. 24606135 10.1146/annurev-biochem-060713-035314 PMC7851832

[21] Rnjak-Kovacina J , Tang F, Whitelock JM et al Silk biomaterials functionalized with recombinant domain V of human perlecan modulate endothelial cell and platelet interactions for vascular applications. Colloids Surf B Biointerfaces 2016;148:130–8. 27591944 10.1016/j.colsurfb.2016.08.039

