# Supplementary material for: Syndecan-4 functionalization of tissue regeneration scaffolds improves interaction with endothelial progenitor cells
Source: Regen Biomater. 2021 Nov 29;8(6):rbab070. doi: 10.1093/rb/rbab070 (PMC8659348; doi:10.1093/rb/rbab070)
Supplement: rbab070_Supplementary_Data [file rbab070_supplementary_data.docx]

Supplementary document


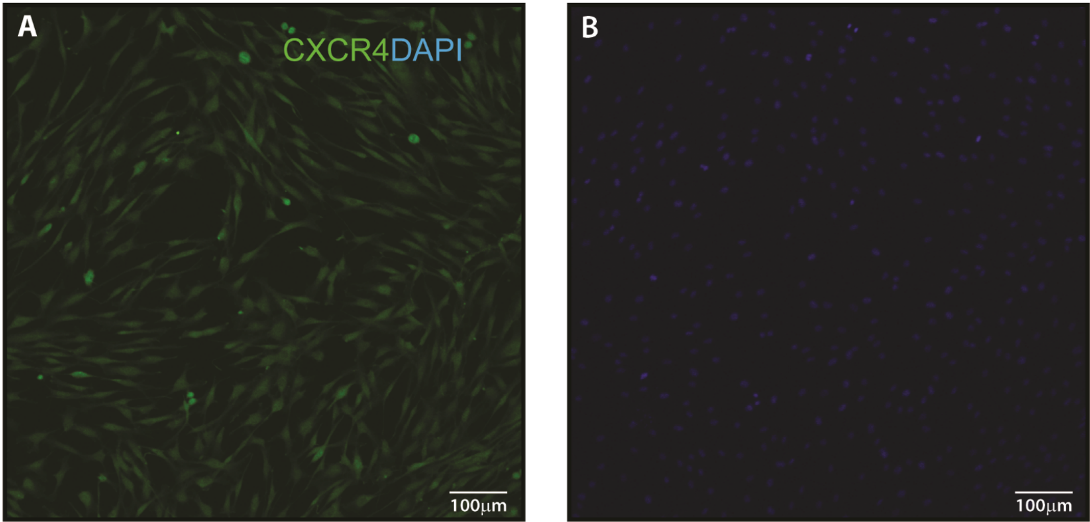


Supplementary Figure 1(A). CXCR4 detection on EPCs. Green staining indicates CXCR4 and blue staining indicates Nuclei. (B) Negative control staining (no anti-CXCR4 antibody). Scale bar = 100 µm.
